# The development of fears of compassion scale Japanese version

**DOI:** 10.1371/journal.pone.0185574

**Published:** 2017-10-12

**Authors:** Kenichi Asano, Masao Tsuchiya, Ikuo Ishimura, Shuzhen Lin, Yuki Matsumoto, Haruko Miyata, Yasuhiro Kotera, Eiji Shimizu, Paul Gilbert

**Affiliations:** 1 Research Center for Child Mental Development, Chiba University, Chiba, Japan; 2 Occupational Stress Research Group, National Institute of Occupational Safety and Health, Kawasaki, Japan; 3 Faculty of Applied Psychology, Tokyo Seitoku University, Tokyo, Japan; 4 Graduate School of Comprehensive Human Sciences, University of Tsukuba, Tsukuba, Japan; 5 Online Learning, University of Derby, Derby, United Kingdom; 6 College of Health and Social Care Research Centre, University of Derby, Derby, United Kingdom; Eberhard-Karls-Universitat Tubingen Medizinische Fakultat, GERMANY

## Abstract

**Objectives:**

Cultivation of compassion is a useful way to treat mental problems, but some individuals show resistance. Fears of compassion can be an obstacle for clinicians when providing psychotherapy, and for clients when engaging in interpersonal relationships. Despite its importance, a Japanese version of fears of compassion scales (for others, from others, and for self) has not yet been developed. This study developed a Japanese version of the Fears of Compassion Scales and tested its reliability and validity.

**Design:**

This study used a cross-sectional design, and a self-report procedure for collecting data.

**Methods:**

A total of 485 students (121 males and 364 females) answered self-report questionnaires, including the draft Fears of Compassion Scales—Japanese version.

**Results:**

There were distinctive factor structures for fear of compassion from others, and for self. The fear of compassion from others scale consisted of concern about compassion from others and avoidance of compassion from others. All scales had good internal consistency, test-retest reliability, face validity, and construct validity. Discrimination and difficulty were also calculated.

**Conclusions:**

These results indicate that the Fears of Compassion Scales—Japanese version is a well-constructed and useful measure to assess fears of compassion and the existence of cultural differences in fears of compassion.

## Introduction

In a little more than a decade, compassion has become a key concept in clinical psychology, particularly in mental health fields, and a several studies have addressed its importance. Macbeth and Gumley conducted a meta-analysis using 14 eligible studies and reported a large effect size for the relationship between self-compassion (compassion toward self) and psychopathology [1]. In clinical research, Gilbert developed compassion focused therapy (CFT), which reinforces compassion toward one’s self and others [2]. Gilbert and Procter showed that compassionate mind training, which is a kind of CFT, reduces depression, anxiety, self-criticism, shame, inferiority, and submissive behaviour in patients with high levels of shame [3]. Gale et al., showed improvements in 73% of people with bulimia nervosa who were treated with CFT [4]. These reports demonstrate the effectiveness and usefulness of developing a compassionate mind.

In CFT, emotional regulation is regarded as the interaction of three motivational systems. The first is the threat and protection system, which detects and copes with threat. The threat and protection system directs attentional focus toward danger and is associated with negative emotions (e.g., anxiety, fear, anger, or disgust). As a result, people tend to take avoidant actions. The second is the seeking and acquiring system, which directs attention toward rewards and resources that support life. This system engenders positive emotions (e.g., joy, achievement, excitement, and vitality). The third is the contentment and soothing system. This system relates to attachment and affiliation, provides well-being and contentment, and can regulate the other two systems if they are overactive. The aim of CFT is to acquire a balanced mind and to regulate emotions by developing a compassionate mind [2].

Training that reinforces compassion was created due to difficulties in implementing this in general cognitive behavioural therapy. Gilbert et al. pointed out that some individuals show resistance to feeling compassion for the self or for others, or receiving compassion from others [5]. Such individuals might have experienced insecurity in their backgrounds and insufficient emotional support from other people. Thus, they tend to have anxious responses to attachment, or avoid and withdraw from others. Individuals who have fear of compassion from others experience certain types of affiliative emotions as more threatening than pleasant [5]. Thus, they may feel fear when the therapist shows affection, kindness, or validation in therapeutic sessions. This can reduce the effectiveness of the care which they receive from significant others or their therapist. To treat and enhance compassion, clinicians need to assess and deal with these blocks or resistances.

Gilbert developed fears of compassion scales, which measure fears of compassion for others, from others, and for self [5]. These self-report scales ask about an individual’s views on compassion and kindness. Individuals with fear of compassion for others regard their affiliative, or compassionate, attitudes and behaviours as more threatening than pleasant because they worry about being used or becoming dependent. Individuals with fear of compassion from others feel fear when they receive compassionate and kind cues and actions. As aforementioned, this tendency can hinder interpersonal relationships, including in daily life and clinical settings. Individuals with fear of compassion for the self tend to criticise themselves. They prone to believe that they are not deserving, and regard having compassion for themselves as a weakness. The fear of compassion for the self can be marked, especially in people with low affection or from abusive backgrounds [6]. Although assessing fears of compassion is necessary and these three aspects of fears of compassion are important in supporting clients and developing their compassion, a Japanese version of Fears of Compassion Scales does not yet exist. Accordingly, this study developed a Japanese version of the Fears of Compassion Scales and assessed the reliability and validity of this tool. To provide this tool with high quality, this research was conducted in procedures based on COnsensus-based Standards for the selection of health Measurement INstruments (COSMIN) checklist [7].

## Materials and methods

### Overview

All procedures were performed in accordance with the Declaration of Helsinki. Required ethical approval was obtained from the ethics committee of Chiba University (No. 1872).

### Preliminary study (Preparation of the Japanese version of the fears of compassion scales)

#### Procedure

To assure cross-cultural validity, procedures were based on a previous guideline [8]. Two psychologists translated three fears of compassion scales into Japanese. II is a clinical psychologist with a PhD, with experience of translation, who has translated a book on self-compassion into Japanese. YM has completed a PhD and worked as a clinical psychologist in Australia. She has a certification for teaching Japanese in Japan. After being translated into Japanese, the questions were back-translated into English. The back-translators were a Singaporean psychologist (SL) and a psychologist from a translation agency. After back-translation, the first author (KA), an epidemiology/psychology researcher (MT), and the translators discussed the Japanese version and the back-translation by comparing it with the original version.

In the next step, the modified Japanese version was back-translated into English by a clinical psychologist who graduated from college in the U.S., and an English psychologist from a translation agency. The author (PG) of the original version and the Portuguese version checked the revised back-translated version. Following this crosscheck, KA, MT, and the translators checked the modified Japanese version again.

Additionally, to confirm face validity, a pilot survey was conducted. Twenty undergraduate students checked and answered the draft of the Japanese version. They were asked to list queries about the questions and to note any difficulties they experienced when completing the draft scales.

#### Results and discussion

The meaning of compassion in Japanese was discussed. Lexically, compassion is translated as ‘Jihi’, which is a Buddhist term. However, this term is used infrequently in Japanese daily life. Therefore, we used ‘Omoiyari’, which means kindness, compassion, or humanity. ‘Omoiyari’ is a casual word that is easy to understand, so that scale items can be used with a wide range of the population.

After review by authors of the original and Portuguese versions of the scale, there were no questions or modifications for one back-translation, but there were some issues related to the other translation. Based on suggestions and further discussion we concluded that the issues were related to the quality of the back-translation. Therefore, we did not modify the integrated Japanese version. In the pilot survey, no questions or suggestions were provided by participants. The face validity of FCS-J was thus ascertained.

### Main survey

#### Procedure

Main surveys were conducted twice, at 3-week intervals, to assess test-retest reliability. All participants were recruited from introductory psychology classes in three Japanese universities. We provided the aim and the informed consent form as the cover of the questionnaire. To maintain anonymity, written consent was not obtained. Participants were asked to answer the questions if they agreed to participate in the study. These procedures were approved by ethics committee of Chiba University.

#### Measurement at time 1

Measurements used at time 1 were Draft of Fears of Compassion Scales—Japanese version (FCS-J), Self-Compassion Scale Japanese version (SCS-J), Multidimensional Perfectionism Cognitions Inventory (MCPI), and Depression Anxiety and Stress Scale (DASS15).

SCS-J is a Japanese version of the Self Compassion Scale (SCS) developed by Neff, which consists of 26-items, and which is the most popular scale used to measure compassion [9]. We used this scale to verify concurrent validity because SCS was used in the development of original version. Both the SCS-J and the SCS have adequate reliability and validity. SCS-J and SCS consist of six factors, which include three positive facets (self-kindness, common humanity, and mindfulness) and three negative facets (self-judgement, isolation, and over-identification). Participants were asked to state how often they acted in the manner stated in each of the items on a scale of 1 (*almost never*) to 5 (*almost always*). In this study, we scored the three positive factors (self-compassion) and three negative factors (self-coldness) independently. This follows the procedure used in the development of the original version, and thus allows for the comparison of cross-cultural differences.

Considering that self-criticism can act as a mediator between unhealthy perfectionism and distress, fears of compassion is related to unhealthy perfectionism [10]. The MCPI was developed to measure cognitions related to both positive and negative self-oriented perfectionism in Japanese individuals [11]. The MCPI consists of three scales (personal standards, pursuit of perfection, and concern over mistakes) with five items each. Participants were asked how often they experienced thoughts related to perfectionism over the last week by responding to each of the items on a scale of 1 (*never*) to 4 (*always*). We scored the three scales independently, particularly to reveal the relationship between fears of compassion and pursuit of perfection and concern over mistakes, the latter of which is thought to be similar to self-criticism. We used this scale as an indicator of concurrent validity

DASS15 is Japanese version of DASS21 [12]. DASS21 is the short version of the Depression Anxiety Stress Scales, which assess depression, anxiety, and stress [13]. The DASS21 consists of three scales with seven items each, but the Japanese version consists of three scales with five items each, based on exploratory and confirmatory factor analysis and internal consistency [14]. We used DASS15 to verify concurrent validity because DASS21 was used in the development of original version. Participants were asked to answer to what extent each of the items applied to them over last week, on a scale of 0 (*did not apply to me at all*) to 3 (*applied to me very much*, *or most of the time*). We scored the three scales independently, to allow comparison of cross-cultural differences with the original version.

#### Measurement at time 2

Measurements used at time 2 were Draft of Fears of Compassion Scales—Japanese version (FCS-J), Japanese version of the Adult Attachment Style Scales for the generalised other (ECR-GO), Sense of Basic Trust Scale and Sense of Interpersonal Trust Scale (SBTS and SITS), and Pros to Vegetable Consumption Behaviour.

ECR-GO is an attachment-style scale for generalised others, which consists of 30 items, split into two factors (anxiety and avoidance) [15]. ECR-GO measures attachment patterns in general relationships, and has good reliability and validity. We used this scale as indicator of concurrent validity. Participants were asked to what extent each item applied to them on a scale of 1 (*did not apply to me at all*) to 7 (*applied to me very much*). We scored the two factors independently to reveal concurrent validity in detail.

SBTS and SITS were developed to measure basic trust [16]. Fear of compassion is thought to be strongly related to trust. People with greater trust in the self and others will show lower fear of compassion. We used these scales as indicators of concurrent validity. SBTS consists of six items, while SITS consists of five items. Both scales have good reliability and validity. Participants were asked to what extent each of the items applied to them. Responses were collected on a scale of 1 (*did not apply to me at all*) to 7 (*applied to me very much*).

To measure pros of vegetable consumption behaviour, the Decisional Balance Scale of vegetable consumption behaviour (DBS) was used [17]. DBS is a scale that measures decisions related to vegetable consumption via two factors, namely pros and cons. The pros factor includes two items, which measure general attitudes toward vegetable consumption. The cons factor also includes two items, which measure attitudes toward not eating vegetables. In this study, only the two items of the cons factor were used to verify discriminant validity with fears of compassion. We used this scale as an indicator of discriminant validity, because we expected it to have no relation to fear of compassion. Participants were asked how important each item is when they make a decision to eat vegetable, on a scale of 1 (*not at all important*) to 5 (*very important*).

We assessed test-retest reliability in individuals via a global rating of change (GRC) assessment. Participants rated to what extent their mind and body had changed on a scale of 1 (*improved very much*) to 7 (*deteriorated very much*). Individuals who considered that they had not changed entered the test-retest analysis.

### Data analysis

Analysis was conducted using R version 3.2.3 [18]. Missing data on all continuous scales were handled using multiple imputation assuming missing at random. Confirmation of the factor structure and item response theory method were conducted by using the date at time 1. Part of concurrent validity were analysed by using the date at time 2. The test-retest reliability was calculated by using the date of participants who responded to both surveys and reported unchanged in GRC.

At first, we conducted confirmatory factor analysis and checked goodness-of-fit indices and factor loadings to examine the factor structures of original scales. When these did not meet the criteria in previous guideline (CFI is not less than .90, RMSEA is not greater than .08, and factor loadings are not less than .32) [19, 20], we explored the factor structures. Specifically, to construct a statistically justifiable factor structure, we verified factor structures with parallel analysis and minimum average partial (to decide number of factors), as well as exploratory factor analyses. In the next step, we conducted confirmatory factor analysis to assess the goodness of fit of hypothesized structures. If two or more factors were suggested in exploratory factor analysis, we considered a bi-factor model and compared the bi-factor model to a multi-factor model in confirmatory factor analysis. Because the bi-factor model can consist of a general factor (that is composed of all items) and group factors, we could explore the possibility of multi-dimensional structures, even when assuming a underlying uni-dimensional structure [21–23]. In cases where we found deleted items or different structures from those in the original version, as a developer of original version, PG checked the factor structures and items to verify content validity.

The item response theory methods of the graded response model were used to confirm the uni-dimensionality of each factor [24]. We evaluated floor and ceiling effects using standards adopted in previous studies, which were based on COSMIN guidelines. These studies defined floor and ceiling effects as when more than 15% of participants generated the minimum or maximum score for each scale [25–29]. Additionally, to evaluate test-retest reliability, intraclass correlations were calculated after participants who responded with a change in GRC were accepted. To evaluate concurrent validity, Pearson’s correlation coefficients were calculated.

## Results

### Age and sex

A total of 260 students (64 males and 196 females) participated in the first survey (time 1). The mean age was 19.34 (SD = 3.26). A total of 225 students (57 males and 168 females) participated in second survey (time 2). The mean age was 19.24 (SD = 3.47). Of these, there were 144 (41 males and 103 females) participants who responded to both surveys. The mean age was 19.29 (SD = 4.27).

### Factor structures and item response theory

In the original factor structure of the compassion for others scale, the indicators of goodness of fit were as follows: CFI = .96, RMSEA = .05, SRMR = .05, and AIC = 97.53. Although the indicators met the criteria, factor loadings for three of the items (‘Being compassionate towards people who have done bad things is letting them off the hook’, ‘People need to help themselves rather than waiting for others to help them’, and ‘For some people, I think discipline and proper punishments are more helpful than being compassionate to them’) were .22, .16, and .17; so, we proceeded to parallel analysis and minimum average partial method to explore and construct a statistically justifiable factor structure. Both results of the parallel analysis and minimum average partial method indicated one factor structure for the compassion for others scale. To confirm the structure of the fears of compassion for others scale, factor analysis (maximum-likelihood method) was conducted. The loadings of three items, as noted above, were less than .32, which was considered sufficiently low that these items were excluded. Factor analysis was conducted again with the remaining seven items. The final factor structure revealed by the exploratory factor analysis, as well as the mean, standard deviation, skewness, and kurtosis of each item, are shown in Table 1. The proportion of variance explained was 46.41% and Cronbach’s α was .79. To test the suitability of the structure suggested by the exploratory factor analysis, we conducted confirmatory factor analysis. The factor loadings of the confirmatory factor analysis, difficulty, and discrimination parameters for all items for each factor, by graded response models, are presented in Table 2. The indicators of goodness of fit were as follows: CFI = .96, RMSEA = .08, SRMR = .05, AIC = 65.62.

**Table 1:** Factor loadings for fears of compassion for others.

No	Item	Factor	Mean	*SD*	skewness	kurtosis
	Fear of Compassion for Others (*α* = .79)					
**5**	People will take advantage of you if you are too forgiving and compassionate	.90	2.51	1.11	-.56	-.51
**4**	I fear that being too compassionate makes people an easy target	.87	2.56	1.07	-.50	-.50
**1**	People will take advantage of me if they see me as too compassionate	.58	2.11	1.05	-.15	-.80
**9**	Being too compassionate makes people soft and easy to take advantage of	.55	1.65	1.08	.31	-.68
**6**	I worry that if I am compassionate, vulnerable people can be drawn to me and drain my emotional resources	.48	2.03	1.14	.07	-1.02
**8**	I fear that if I am compassionate, some people will become too dependent upon me	.38	2.39	.92	-.37	-.10
**3**	There are some people in life who don’t deserve compassion	.36	2.21	1.29	-.17	-1.15
	Variance explained (%)	46.41				

**Table 2:** Confirmatory factor analysis and item response theory outcome for fear of compassion for others.

No	Item	Factor	*a*	*b0*	*b1*	*b2*	*b3*
**5**	People will take advantage of you if you are too forgiving and compassionate	.90	2.62	-1.76	-.84	-.28	.99
**4**	I fear that being too compassionate makes people an easy target	.87	2.16	-2.00	-.96	-.27	.97
**1**	People will take advantage of me if they see me as too compassionate	.58	.80	-2.53	-.77	.36	2.34
**9**	Being too compassionate makes people soft and easy to take advantage of	.55	.77	-1.84	.02	1.15	2.76
**6**	I worry that if I am compassionate, vulnerable people can be drawn to me and drain my emotional resources	.48	.62	-2.83	-.50	.52	2.45
**8**	I fear that if I am compassionate, some people will become too dependent upon me	.38	.50	-4.32	-2.25	.06	3.02
**3**	There are some people in life who don’t deserve compassion	.36	.46	-2.93	-.98	.16	2.09

*a* = Discrimination; *b* = Difficulty

In the original factor structure of the compassion from others scale, the indicators of goodness of fit were as follows: CFI = .75, RMSEA = .15, SRMR = .08, and AIC = 508.08. So, we proceeded to parallel analysis and minimum average partial method to explore and construct a statistically justifiable factor structure. Both results of the parallel analysis and minimum average partial method indicated a two-factor structure of the compassion from others scale. To confirm the structure of the fears of compassion from others scale, factor analysis (maximum-likelihood method, promax rotation) was conducted. The loadings of five items were less than .32. Excluded items were ‘Wanting others to be kind to oneself is a weakness’, ‘I fear that when I need people to be kind and understanding they won’t be’, ‘I’m fearful of becoming dependent on the care from others because they might not always be available or willing to give it’, and ‘I worry that people are only kind and compassionate if they want something from me’. Additionally, an item that loaded on more than one factor with loadings greater than .30 was excluded (‘When people are kind and compassionate towards me I feel empty and sad’). Factor analysis was conducted again with the remaining eight items. The first factor was Concern about Compassion from Others (CCfrO), and second was Avoidance of Compassion from Others (ACfrO). The final factor structure from the exploratory factor analysis, and the mean, standard deviation, skewness, and kurtosis of each item are listed in Table 3. The proportion of variation explained was 68.34% and Cronbach’s α was .85 for the scale as a whole, and .83 and .82 for each of the factors.

**Table 3:** Factor loadings for fear of compassion from others.

No	Item	Factor 1	Factor 2	Mean	*SD*	skewness	kurtosis
	Fear of Compassion from Others (*α* = .85)						
	Concern about Compassion from Others (*α* = .83)						
**5**	Feelings of kindness from others are somehow frightening	.93	.00	1.37	1.15	.68	-.35
**6**	When people are kind and compassionate towards me I feel anxious or embarrassed	.90	.03	1.30	1.12	.72	-.16
**4**	I often wonder whether displays of warmth and kindness from others are genuine	.68	-.01	2.17	1.26	-.29	-1.01
**7**	If people are friendly and kind I worry they will find out something bad about me that will change their mind	.48	.01	2.20	1.12	-.20	-.97
	Avoidance of Compassion from Others (*α* = .82)						
**12**	I try to keep my distance from others even if I know they are kind	-.08	.93	1.04	.99	.99	.71
**13**	If I think someone is being kind and caring towards me, I ‘put up a barrier’	-.03	.87	1.15	1.02	.86	.45
**11**	Even though other people are kind to me, I have rarely felt warmth from my relationships with others	.09	.60	.73	.86	1.48	2.72
**10**	If people are kind I feel they are getting too close	.28	.45	1.06	1.08	.91	.07
	Variance explained (%)	68.34					

To test the suitability of the structure suggested in the exploratory factor analysis, we conducted confirmatory factor analysis and compared goodness of fit between a multifactor model and a bi-factor model (assuming a general factor and two group factors). In the multifactor model, all path coefficients were significant, and the indicators of goodness of fit were as follows: CFI = .96, RMSEA = .09, SRMR = .06, AIC = 92.776. In bi-factor model, all path coefficients were significant and the indicators of goodness of fit were as follows: CFI = .98, RMSEA = .06, SRMR = .03, AIC = 107.19. The factor loadings from the confirmatory factor analysis, difficulty, and discrimination parameters for all items, by graded response models, are presented in Table 4.

**Table 4:** Confirmatory factor analysis and item response theory in fear of compassion from others.

No	Item	General Factor	Specific Factor 1	Specific Factor 2	*a*	*b0*	*b1*	*b2*	*b3*
**5**	Feelings of kindness from others are somehow frightening	.78	.52		2.98	-.74	.36	.95	1.66
**6**	When people are kind and compassionate towards me I feel anxious or embarrassed	.77	.49		2.27	-.70	.41	1.11	1.80
**4**	I often wonder whether displays of warmth and kindness from others are genuine	.47	.51		.94	-1.67	-.67	.07	1.59
**7**	If people are friendly and kind I worry they will find out something bad about me that will change their mind	.39	.28		.58	-1.92	-.29	.57	1.86
**12**	I try to keep my distance from others even if I know they are kind	.52		.73	1.57	-.53	.81	1.57	2.28
**13**	If I think someone is being kind and caring towards me, I ‘put up a barrier’	.52		.66	1.60	-.68	.62	1.51	2.14
**11**	Even though other people are kind to me, I have rarely felt warmth from my relationships with others	.56		.38	.83	-.18	1.84	2.57	3.38
**10**	If people are kind I feel they are getting too close	.64		.26	.87	-.52	.96	1.71	2.94

*a* = Discrimination; *b* = Difficulty

In the original factor structure of the compassion for self scale, the indicators of goodness of fit were as follows: CFI = .81, RMSEA = .12, SRMR = .07, and AIC = 508.12. So, we proceeded to parallel analysis and minimum average partial method to explore and construct a statistically justifiable factor structure. Both results of the parallel analysis and minimum average partial method indicated a two-factor structure of the compassion from others scale. To confirm the structure of the fears of compassion from others scale, factor analysis (maximum-likelihood method, promax rotation) was conducted. The loadings of the item ‘Getting on in life is about being tough rather than compassionate’ were all less than .32 and we dropped it. Additionally, one item with loadings greater than .30 on more than one factor was excluded (‘I have never felt compassion for myself, so I would not know where to begin to develop these feelings’). Factor analysis was conducted again with the remaining 13 items. The first factor was Miserable with Self-Compassion (MSC) and second was Demerits of Self-Compassion (DSC). The final factor structure from the exploratory factor analysis, and the mean, standard deviation, skewness, and kurtosis of each item are shown in Table 5. The proportion of variance explained was 58.88% and Cronbach’s was .91 for the scale as a whole, and .85 and .88 for each factor.

**Table 5:** Factor loadings for fears of compassion for self.

No	Item	Factor 1	Factor 2	Mean	*SD*	skewness	kurtosis
	Fear of Compassion for Self (*α* = .91)						
	Miserable with Self-Compassion (*α* = .85)						
**5**	When I try and feel kind and warm to myself I just feel kind of empty	.85	.01	.95	1.05	1.07	.49
**2**	If I really think about being kind and gentle with myself it makes me sad	.84	-.09	1.07	1.05	.94	.32
**6**	I fear that if I start to feel compassion and warmth for myself, I will feel overcome with a sense of loss/grief	.74	.05	.88	1.06	1.35	1.40
**4**	I would rather not know what being ‘kind and compassionate to myself’ feels like	.70	-.07	.82	.87	1.00	.76
**1**	I feel that I don’t deserve to be kind and forgiving to myself	.44	.23	1.38	1.03	.57	-.10
	Demerit of Self-Compassion (*α* = .88)						
**11**	I fear that if I become too compassionate to myself I will lose my self-criticism and my flaws will show	-.20	.84	2.24	1.30	-.37	-.98
**12**	I fear that if I develop compassion for myself, I will become someone I do not want to be	-.05	.80	1.87	1.27	.12	-1.02
**13**	I fear that if I become too compassionate to myself others will reject me	-.02	.71	1.70	1.21	.11	-1.07
**7**	I fear that if I become kinder and less self-critical to myself then my standards will drop	.08	.67	1.64	1.35	.28	-1.24
**10**	I worry that if I start to develop compassion for myself I will become dependent on it	.15	.62	1.53	1.29	.37	-1.05
**15**	I fear that if I am too compassionate towards myself, bad things will happen	.25	.56	1.70	1.19	.19	-.90
**8**	I fear that if I am more self compassionate I will become a weak person	.24	.55	1.32	1.25	.65	-.73
**14**	I find it easier to be critical towards myself rather than compassionate	.06	.45	2.65	1.23	-.61	-.67
	contribution (%)	58.88					

To test the suitability of the structure suggested in the exploratory factor analysis, we conducted confirmatory factor analysis and compared the goodness of fit between a multifactor model and a bi-factor model (assuming a general factor and two group factors). In the multifactor model, all path coefficients were significant and the indicators of goodness of fit were as follows: CFI = .89, RMSEA = .10, SRMR = .06, AIC = 296.81. In the bi-factor model, all path coefficients were significant and the indicators of goodness of fit were as follows: CFI = .95, RMSEA = .08, SRMR = .04, AIC = 212.83. The factor loadings for the confirmatory factor analysis, difficulty, and discrimination parameters of all items by graded response models are presented in Table 6.

**Table 6:** Confirmatory factor analysis and item response theory outcomes for fears of compassion for self.

No	Item	General Factor	Specific Factor 1	Specific Factor 2	*a*	*b0*	*b1*	*b2*	*b3*
**5**	When I try and feel kind and warm to myself I just feel kind of empty	.49	.69		1.75	-.22	.82	1.45	2.22
**2**	If I really think about being kind and gentle with myself it makes me sad	.33	.75		1.20	-.53	.82	1.56	2.44
**6**	I fear that if I start to feel compassion and warmth for myself, I will feel overcome with a sense of loss/grief	.54	.56		1.40	-.14	1.03	1.66	2.12
**4**	I would rather not know what being ‘kind and compassionate to myself’ feels like	.33	.56		.91	-.29	1.31	2.50	3.59
**1**	I feel that I don’t deserve to be kind and forgiving to myself	.48	.42		.88	-1.25	.35	1.67	2.67
**11**	I fear that if I become too compassionate to myself I will lose my self-criticism and my flaws will show	.43		.50	.90	-1.60	-.82	-.01	1.41
**12**	I fear that if I develop compassion for myself, I will become someone I do not want to be	.45		.63	1.18	-1.26	-.28	.59	1.50
**13**	I fear that if I become too compassionate to myself others will reject me	.40		.59	.97	-1.21	-.13	.74	2.27
**7**	I fear that if I become kinder and less self-critical to myself then my standards will drop	.87		.15	1.45	-.79	.11	.52	1.56
**10**	I worry that if I start to develop compassion for myself I will become dependent on it	.61		.40	1.13	-.82	.17	.79	1.90
**15**	I fear that if I am too compassionate towards myself, bad things will happen	.43		.67	1.22	-1.16	-.12	.78	1.92
**8**	I fear that if I am more self compassionate I will become a weak person	.80		.22	1.52	-.54	.44	.91	1.84
**14**	I find it easier to be critical towards myself rather than compassionate	.30		.40	.60	-2.94	-1.61	-.55	.95

*a* = Discrimination; *b* = Difficulty

The content validity of the three Japanese versions was verified by PG, who developed the original version.

### Floor and ceiling effect

The percentage of participants who scored the minimum was 0% for fear of compassion for others, 1.9% for fear of compassion from others, and 2.6% for fear of compassion for self. The percentage of participants who scored the maximum was 0.3% for fear of compassion for others, 0% for fear of compassion from others, and 0% for fear of compassion for self.

#### Test-retest reliability

To evaluate test-retest reliability, intraclass correlations were calculated for participants whose answers were unchanged in the GRC. Eighty-five participants (25 males and 60 females) answers remained unchanged, mean age was 19.66 (SD = 5.44). The intraclass correlations were .73 (0.59–0.82) in FCfO, .58 (.42-.70) in FCfrO, .64 (0.49–0.75) in CCfrO, .60 (0.45–0.72) in ACfrO, .78 (.67-.85) in FCS, .63 (0.48–0.74) in MSC and .75 (64-.83) in DSC.

### Concurrent validity

Descriptive statistics and Cronbach’s *α* of all variables are shown in Table 7. The correlations between factors of the three fears of compassion scales and SCS-J, MCPI, DASS15, ECR-GO, SBTS, SITS, and DBS were calculated, as shown in Table 8.

**Table 7:** Descriptive statistics and Cronbach’s α of all variables.

Variables	Mean	*SD*	*α*
**Fear of Compassion for Others**	15.44	5.16	.79
**Fear of Compassion from Others**	10.68	6.18	.85
**Concern about Compassion from Others**	6.74	3.95	.83
**Avoidance of Compassion from Others**	3.94	3.16	.82
**Fear of Compassion for Self**	19.66	10.48	.91
**Miserable with Self-Compassion**	5.05	3.96	.85
**Demerit of Self-Compassion**	14.60	7.44	.88
**DASS-15**			
**Depression**	2.70	3.16	.71
**Anxiety**	1.99	2.73	.84
**Stress**	5.23	3.37	.76
**Self-Compassion Scale**	71.84	13.49	.78
**Self-Compassion (Overall)**			
**Self-kindness**	12.54	3.49	.77
**Self-judgement**	15.31	4.25	.78
**Common Humanity**	10.92	3.26	.67
**Isolation**	12.38	4.10	.83
**Mindfulness**	11.77	3.02	.70
**Over identification**	13.73	3.47	.77
**Multidimensional Perfectionism Cognition**			
**Personal Standards**	12.13	4.02	.88
**Pursuit of Perfection**	10.77	4.32	.66
**Concern over Mistakes**	13.07	4.03	.86
**Adult Attachment Style Scale**			
**Anxiety**	65.52	17.77	.90
**Avoidance**	48.16	6.58	.70
**Sense of Basic Trust Scale**	23.66	6.53	.77
**Sense of Interpersonal Trust Scale**	24.03	4.85	.79
**Pros of Vegetable Consumption Behavior**	2.80	1.41	-

**Table 8:** Correlations between factors of three fear of compassion scales and variables.

Measures in Time 1 (N = 260)	FCfO	FCfrO	CCfrO	ACfrO	FCS	MSC	DSC
**Depression**	.37**	.42**	.42**	.31**	.43**	.50**	.34**
**Anxiety**	.24**	.30**	.30**	.22**	.25**	.27**	.20**
**Stress**	.18**	.30**	.36**	.14*	.37**	.36**	.32**
**Self-Compassion (Overall)**	-.15*	-.25**	-.40**	.01	-.47**	-.38**	-.45**
**Self-kindness**	.02	-.09	-.17**	.04	-.29**	-.27**	-.27**
**Self-judgement**	.26**	.36**	.46**	-.13*	.52**	.43**	.51**
**Common Humanity**	.09	.23**	.17**	.24**	.06	.12	.03
**Isolation**	.22**	.37**	.50**	.11	.49**	.41**	.44**
**Mindfulness**	.10	.14*	.09	.16*	.02	-.03	-.01
**Over-identification**	.21**	.33**	.44**	.09	.40**	.31**	.39**
**Personal Standards**	.07	.17**	.13*	.18**	.12	.11	.11
**Pursuit of Perfection**	.21**	.30**	.27**	.24**	.40**	.36**	.37**
**Concern over Mistakes**	.30**	.41**	.46**	.22**	.49**	.40**	.48**
**Anxiety**	.32**	.45**	.51**	.26**	.54**	.45**	.49**
**Avoidance**	.32**	.41**	.36**	.36**	.43**	.41**	.40**
**Sense of Basic Trust Scale**	-.33**	-.50**	-.55**	-.31**	-.58**	-.47**	-.54**
**Sense of Interpersonal Trust Scale**	-.39**	-.55**	-.50**	-.48**	-.38**	-.35**	-.34**
**Pros of Vegetable Consumption Behavior**	.17*	.11	.07	.12	.04	.03	.06

FCfO = Fear of Compassion for Others, FCfrO = Fear of Compassion from Others, CCfrO = Concern about Compassion from Others, ACfrO = Avoidance of Compassion from Others, FCS = Fear of Compassion for Self, MSC = Miserable with Self-Compassion, DSC = Demerit of Self- Compassion.

**p < .01,

*p < .05

All scales of FCS-J and its subscales were correlated with mental health. Particularly in relation to depression, all effect sizes (*r*) were over .30. Regarding fears of compassion from others, FCfrO and CCfrO showed larger effect sizes relative to Isolation, but ACfrO did not. With respect to self-compassion, effect sizes (*r*) between FSC and self-compassion were relatively large. FCS also showed larger effect sizes (*r*) in relation to variables associated with self-criticism (self-judgement and concern over mistakes). For variables pertaining to interpersonal relationships (attachment style and sense of trust), almost all scales and subscales showed effect sizes (*r*) of medium to large magnitude. Pros of vegetable consumption behaviour showed small or nonsignificant correlations with FCS-J.

## Discussion

### Face validity

In this study, we developed a Japanese version of the Fears of Compassion Scales. We conducted translation procedures based on Beaton et at., who recommended two independent translations in order to assure cross-cultural validity [8]. After some discussion, we chose to use the common term ‘omoiyari’, in lieu of its more formal counterpart ‘jihi’, in the draft of the Japanese version of the scale. In stage 4, our scales were reviewed by the developer of the original version of the scale (PG) and the Portuguese version, to verify face validity. In stage 5, we conducted a primary survey to verify face validity, which revealed no items with particular issues or difficulties. These procedures suggest that the FCS-J has face validity.

### Factor structure

Ten items were excluded and alternative structures were investigated for three scales. For the fear of compassion for others scale, on the basis of low factor loadings we excluded ‘Being compassionate towards people who have done bad things is letting them off the hook’, ‘People need to help themselves rather than waiting for others to help them’, and ‘For some people, I think discipline and proper punishments are more helpful than being compassionate to them’. These items seem to represent beliefs or values about showing compassion and punishment, rather than emotions related to fear. With respect to items with low factor loadings on the fear of compassion from others scale, ‘Wanting others to be kind to oneself is a weakness’ appears to be a value judgment with respect to strength and weakness. ‘I fear that when I need people to be kind and understanding they won’t be’ and ‘I’m fearful of becoming dependent on the care from others because they might not always be available or willing to give it’ may be interpreted as situations without directed compassion from others, and ‘I worry that people are only kind and compassionate if they want something from me’ appears to be a belief about kindness rather than an emotion related to fear. Additionally, ‘When people are kind and compassionate towards me I feel empty and sad’ can be considered to reflect depression or sadness rather than fear or anxiety, and thus was related to both sub factors. In the fear of compassion for self scale, ‘Getting on in life is about being tough rather than compassionate’ appears to be also a belief regarding toughness and showed low factor loadings. ‘I have never felt compassion for myself, so I would not know where to begin to develop these feelings’ might be understood more as confusion than an emotion related to fear, and thus loaded on both sub factors.

Throughout, excluded items were considered reflective of values or beliefs rather than emotions related to fear. This result might relate to East Asian culture. In that culture, shame is a key emotion that is tied to the fear of rejection by the community [30]. In cultural anthropology, Benedict noted that shame and related inter-personal emotions are important in Japanese society [31]. Some studies suggest that Japanese people have higher public self-consciousness than people in western cultures [32]. In Japanese culture, sympathizing with other people is more important than one’s own values, and values have higher situational dependency than others’ opinions or the implicit context. In contrast, the emotion of fear of rejection is strongly rooted; thus, only items that reflect fear might form factors. Although our Japanese scales adopted a bi-factor model and included original factor structures, further surveys and studies are necessary in order to compare cultural differences in the structure of fear of compassion and shame, by analysing differential item functioning from data obtained in other countries.

In the subfactor of FCfrO, one factor reflects the emotional (fear, anxiety, or shame) and cognitive (worry) response, and the other factor reflects distancing the self from compassion from others. These two factors form the uni-dimensional FCfrO. This structure is consistent with attachment theory (avoidance and anxiety).

In the subfactor FCS, one factor reflects the emotional response to having compassion for the self, as represented by a sense of loss. The other factor reflects the cognitive response to having compassion for the self, as represented by anticipatory anxiety or worry.

Differences in factor structures and item deletion are not unusual in cross-cultural studies, as some reports have shown cultural bias in the factor structure and item levels [31–36]. To identify causes of the differences, cross-cultural surveys will be required, using differential item functioning analysis.

### Floor and ceiling effects

Although the floor and ceiling effects did not exist, the mean scores of each scale were low, considering the possible score ranges. However, this tendency was also found in data obtained from student samples in previous studies, and is likely due to recruiting healthy individuals [5][37]. Clinical samples should be assessed in future studies to reveal more detail regarding the responsiveness of the Fears of Compassion Scale Japanese Version.

### Internal consistency, item response theory (IRT), and test re-test reliability

The internal consistency of the 3 scales in the original version were .85, .91, .94 according to Gilbert et al. [37]. In our Japanese version, internal consistency was over .80 in all factors and scales. IRT methodology also revealed enough discrimination in all scales. Intraclass correlations of test-retest reliability were enough (from .58 to .78.). Although IRT and test-retest reliability of original version have not been examined, the FCS-J has enough reliability in internal consistency, IRT, and test re-test reliability.

### Strengths and limitations

The first strength of this study is that we successfully developed the Fears of Compassion Scale Japanese version (FCS-J) based on COSMIN guidelines. COSMIN guidelines ensure the quality of research outcomes. By using these guidelines, FOCS-J is likely highly reliable and valid, despite its limitations.

The second strength relates to clinical benefits. As mentioned earlier, compassion has a significant role in mental health, and developing compassion is helpful for effective treatment. However, when clients show the resistance, fear, or confusion toward developing compassion, the therapist cannot develop compassion in the client without addressing these roadblocks. The concept of fear of compassion encompasses problems with developing compassion in clinical settings. This includes fear, resistance, or anxiety of having compassion for self, receiving compassion from others, or have compassion for others.

Those with fear of compassion for others tend to regard receiving help as a weakness, and they are also afraid of being used. Thus, they avoid intimate interpersonal relationships because of suspicion. This tendency relates to attachment style and self-interest; the therapist can assess attitudes toward others or trust of others [5]. If clients have high fear of compassion for others, they might have a greater prevalence of explicit interpersonal problems, such as arguments or avoiding relationships.

Those with fear of compassion from others often have difficulties forming a relationship with a therapist in a clinical setting. In psychotherapy, the therapist shows understanding of the client’s distress in a warm and caring manner. Such attitudes are usually a bridge to exchange thoughts, feelings, ideas, or impressions, but individuals with difficulties in receiving compassion from the therapist will feel anxiety and be suspicious of the therapist’s response. These are significant problems in therapy; FCS-J may help the therapist to understand how their clients can rely on others. Indeed, fear of compassion from others moderates the relationship between depression and self-criticism [38]. After providing clients with stable affiliation and warmth, the former will learn mutual trust, and accept the development of compassion. Such an intervention will relieve the individual from self-critical inner thoughts because these thoughts will be displaced by the therapist’s warmth.

People with fear of compassion for the self exhibit high self-criticism. Fear of compassion for the self is to not accept oneself and to punish oneself if one’s demands are not met. Previous studies have shown that individuals with fear of compassion for the self regard themselves as undeserving, and they have an unsatisfied desire to be affirmed and accepted. Such a state is often developed in the context of maltreatment and abuse; thus, the therapist must pay attention to the client’s self-criticism and history of trauma.

Even though compassion for the self is key in improving mental problems, it is cultivated by receiving compassion from others and showing compassion toward others. Attachment consists of the need to be loved and cared for, and to cherish ourselves. Showing compassion toward others will lead others to give us compassion. All three perspectives interact and the therapist must enhance all in a balanced manner. All of the scales developed in this study will help therapists to understand their clients’ state of compassion, including interpersonal style, trust, and kindness.

However, there are some limitations to this study. One limitation of this study is that we sampled only Japanese students. Although the samples used in the development of the original version were university students and psychotherapists, it is advisable to re-examine our scale development using a more representative sample of the population, including clinical and non-clinical groups. This would enhance the generalizability, and could discover other functions of fears of compassion. Because the structures of the Japanese scales differ from those of the original version, there is a need for cross-cultural studies on differential item functions, using full items (S1 and S2 Appendices). Similarly, evaluating differential item functions is needed to help elucidate psychological pathology. This could enable patients to receive appropriate interventions. In addition, this study used a cross-sectional design and therefore cannot reveal causal inferences. Because fears of compassion is a key aspect of CFT or other psychotherapies, assessing changes in fears of compassion between pre- and post-intervention periods could provide clinicians with important suggestions with respect to treatment resistance, reoccurrence, therapeutic relationships, and so forth.

## Conclusion

In this study, we developed FCS Japanese version. To provide strong reliability and validity, we added some translation procedures and psychometric analysis (2 way translations, parallel analysis, minimum average partial, confirmatory factor analysis, item response theory and test-retest method). The results indicated differences in factor structures of FCfrO and FCS that are presumed to be related to cultural differences. The indicators of goodness of fit in confirmatory factor analysis, internal consistency, test-retest reliability, and difficulty and discriminant parameters in item response theory were good or enough. Construct validities are also good and the relations between fears of compassion and self-compassion, unhealthy perfectionism, attachment style, basic trust and interpersonal trust.

## Supporting information

S1 Appendix Fears of compassion scales Japanese version. (DOCX)

S2 Appendix Fears of compassion scales. (PDF)

S1 Table Datasets. (XLSX)
