# Supplementary material for: The development of fears of compassion scale Japanese version
Source: PLoS One. 2017 Oct 12;12(10):e0185574. doi: 10.1371/journal.pone.0185574 (PMC5638239; doi:10.1371/journal.pone.0185574)
Supplement: S1 Appendix — (DOCX) [file pone.0185574.s001.docx]

**慈悲に対する恐怖尺度日本語版**

**Fears of Compassion Scale Japanese version**

『思いやり』や『やさしさ』に対する考えはいろいろです。「いつでもどんなときにも人を思いや

り、やさしくすることは大切だ」と考える人もいますし、「をえた思いやりややさしさはいつも良いとはかぎらない」と考える人もいます。以下の3分野のそれぞれの質問について、あなたのお考えがどのくらい当てはまるかを聞かせてください。

Ａ．人を思いやる・やさしくする

Ｂ．人から思いやりをうける・やさしくされる

Ｃ．自分を思いやる・やさしくする

各文の内容が自分にどのくらい当てはまるか、その度合いを表す数字0~4の一つに○をしてください。

どれくらい当てはまるかという度合いは、次のようになります。

全く当てはまらない　　0　　1　　2　　3　　4　　とてもよく当てはまる

0「全く当てはまらない」～4「に当てはまる」の間の

1、2、3は、いくらか当てはまるということになります。

質問Ａ：人を思いやる・やさしくする

| 1 | 私がやさしすぎる人間だとわかれば、人は私を利用しようとするだろう。 | 0 　1 　2 　3 　4 |
| --- | --- | --- |
| 2 | 悪いことをした人に思いやりを示すと、その人を許してしまうことになる。 | 0 　1 　2 　3 　4 |
| 3 | 世の中には、思いやる価値のない人もいる。 | 0 　1 　2 　3 　4 |
| 4 | 思いやりが強すぎる人は、いいように利用されると思う。 | 0 　1 　2 　3 　4 |
| 5 | さや思いやりが強すぎると、人に利用されてしまう。 | 0 　1 　2 　3 　4 |
| 6 | 思いやり深いと、弱い人に取りまかれてしまい、しんどくなると思う。 | 0 　1 　2 　3 　4 |
| 7 | 人が助けてくれるのを待っているのではなく、自分で自分を助ける方が大切だと思う。 | 0 　1 　2 　3 　4 |
| 8 | もし私が思いやりのある人だと、私に頼りすぎる人もでてくると思う。 | 0 　1 　2 　3 　4 |
| 9 | 思いやりが深すぎる人は、弱く、利用されやすい。 | 0 　1 　2 　3 　4 |
| 10 | 思いやりよりも、しつけや適度なのほうが、その人のためになることもある。 | 0 　1 　2 　3 　4 |

質問Ｂ：人から思いやりをうける・やさしくされる

| 1 | 他人の親切をするのは、一種の弱さである。 | 0 　1 　2 　3 　4 |
| --- | --- | --- |
| 2 | 親切にしてほしい理解してほしいと思っても、そうしてくれる人はいないと思う。 | 0 　1 　2 　3 　4 |
| 3 | いつも気にかけてくれるとは限らないので、人をりにするのは不安だ。 | 0 　1 　2 　3 　4 |
| 4 | 人が示すかさや思いやりは本物だろうかとたびたび考える。 | 0 　1 　2 　3 　4 |
| 5 | 人から優しくされると、何となく怖くなる。 | 0 　1 　2 　3 　4 |
| 6 | 人から親切にされたり思いやりを示されたりすると、不安や戸惑いを感じる。 | 0 　1 　2 　3 　4 |
| 7 | 友情や思いやりを示してくれる人が、私のを知り、心変わりしてしまうのが怖い。 | 0 　1 　2 　3 　4 |
| 8 | 何か私にしてほしいことがあるときだけ、人は私にやさしくしてくる。 | 0 　1 　2 　3 　4 |
| 9 | 人のやさしさや思いやりを感じると、むなしくなり悲しくなる。 | 0 　1 　2 　3 　4 |
| 10 | 人にやさしくされると、その人との関係が近くなりすぎているように感じる。 | 0 　1 　2 　3 　4 |
| 11 | やさしくされても、その人との関係にかさを感じたりすることはほとんどない。 | 0 　1 　2 　3 　4 |
| 12 | その人がやさしい人だと分かっていても、距離をおくようにしている。 | 0 　1 　2 　3 　4 |
| 13 | やさしく世話してくれているとわかっていても、その人との間に壁を作る。 | 0 　1 　2 　3 　4 |

質問C：自分を思いやる・やさしくする

| 1 | 私は自分にやさしくしたり、自分を許したりするような価値はないと思う。 | 0 　1 　2 　3 　4 |
| --- | --- | --- |
| 2 | 自分にほんとうにやさしくしようと考えると悲しくなる。 | 0 　1 　2 　3 　4 |
| 3 | 生きていくためには、やさしさよりも強さが必要だ。 | 0 　1 　2 　3 　4 |
| 4 | 自分にやさしいとか自分を思いやることがどういうことなのか、分かりたくもない。 | 0 　1 　2 　3 　4 |
| 5 | 自分にやさしくしようと思うと、ただむなしい気持ちになる。 | 0 　1 　2 　3 　4 |
| 6 | 自分に思いやりを感じ始めたら、や深い悲しみに押しつぶされそうでこわい。 | 0 　1 　2 　3 　4 |
| 7 | 自分にやさしく甘くなったら、人としてのが下がってしまいそうで心配だ。 | 0 　1 　2 　3 　4 |
| 8 | 自分に対する思いやりが深くなれば、弱い人間になるのではないかと心配だ。 | 0 　1 　2 　3 　4 |
| 9 | 自分を思いやることがないので、どうすればそういう気持ちになれるのか分からない。 | 0 　1 　2 　3 　4 |
| 10 | 自分を思いやるようになりだしたら、いつもそうしてしまうのではないかと心配だ。 | 0 　1 　2 　3 　4 |
| 11 | 自分に対する思いやりの度がすぎると、に自分を見なくなり、欠点がでてくると思う。 | 0 　1 　2 　3 　4 |
| 12 | 自分に対する思いやりが当たり前になると、自分がなりたくない人になってしまうと思う。 | 0 　1 　2 　3 　4 |
| 13 | 自分に対する思いやりが深くなりすぎると、人から受け入れられなくなると思う。 | 0 　1 　2 　3 　4 |
| 14 | 自分を思いやるよりする方が、だと思う。 | 0 　1 　2 　3 　4 |
| 15 | 自分に対する思いやりが深くなりすぎると、何か悪いことがおこりそうでこわい。 | 0 　1 　2 　3 　4 |
