# Supplementary material for: The development of fears of compassion scale Japanese version
Source: PLoS One. 2017 Oct 12;12(10):e0185574. doi: 10.1371/journal.pone.0185574 (PMC5638239; doi:10.1371/journal.pone.0185574)
Supplement: S2 Appendix — (PDF) [file pone.0185574.s002.pdf]

FEARS OF COMPASSION SCALES

Different people have different views of compassion and kindness. While some people believe that it is important to show compassion and kindness in all situations and contexts, others believe we should be more cautious and can worry about showing it too much to ourselves and to others. We are interested in your thoughts and beliefs in regard to kindness and compassion in three areas of your life:

1. Expressing compassion for others
2. Responding to compassion from others
3. Expressing kindness and compassion towards yourself

Below are a series of statements that we would like you to think carefully about and then circle the number that best describes how each statement fits you.

SCALE

Please use this scale to rate the extent that you agree with each statement

Don't agree at all	0	1	2	3	4	Completely agree
			Somewhat agree			

Scale 1: Expressing compassion for others

1.	People will take advantage of me if they see me as too compassionate	0	1	2	3	4
2.	Being compassionate towards people who have done bad things is letting them off the hook	0	1	2	3	4
3.	There are some people in life who don't deserve compassion	0	1	2	3	4
4.	I fear that being too compassionate makes people an easy target	0	1	2	3	4
5.	People will take advantage of you if you are too forgiving and compassionate	0	1	2	3	4
6.	I worry that if I am compassionate, vulnerable people can be drawn to me and drain my emotional resources	0	1	2	3	4
7.	People need to help themselves rather than waiting for others to help them	0	1	2	3	4
8.	I fear that if I am compassionate, some people will become too dependent upon me	0	1	2	3	4
9.	Being too compassionate makes people soft and easy to take advantage of	0	1	2	3	4
10.	For some people, I think discipline and proper punishments are more helpful than being compassionate to them	0	1	2	3	4

Scale 2: Responding to the expression of compassion from others

1.	Wanting others to be kind to oneself is a weakness	0	1	2	3	4
2.	I fear that when I need people to be kind and understanding they wont be	0	1	2	3	4
3.	I'm fearful of becoming dependent on the care from others because they might not always be available or willing to give it	0	1	2	3	4
4.	I often wonder whether displays of warmth and kindness from others are genuine	0	1	2	3	4
5.	Feelings of kindness from others are somehow frightening	0	1	2	3	4
6.	When people are kind and compassionate towards me I feel anxious or embarrassed	0	1	2	3	4
7.	If people are friendly and kind I worry they will find out something bad about me that will change their mind	0	1	2	3	4
8.	I worry that people are only kind and compassionate if they want something from me	0	1	2	3	4
9.	When people are kind and compassionate towards me I feel empty and sad	0	1	2	3	4
10.	If people are kind I feel they are getting too close	0	1	2	3	4
11.	Even though other people are kind to me, I have rarely felt warmth from my relationships with others	0	1	2	3	4
12.	I try to keep my distance from others even if I know they are kind	0	1	2	3	4
13.	If I think someone is being kind and caring towards me, I 'put up a barrier'	0	1	2	3	4

Scale 3: Expressing kindness and compassion towards yourself

1.	I feel that I don’t deserve to be kind and forgiving to myself	0	1	2	3	4
2.	If I really think about being kind and gentle with myself it makes me sad	0	1	2	3	4
3.	Getting on in life is about being tough rather than compassionate	0	1	2	3	4
4.	I would rather not know what being ‘kind and compassionate to myself’ feels like	0	1	2	3	4
5.	When I try and feel kind and warm to myself I just feel kind of empty	0	1	2	3	4
6.	I fear that if I start to feel compassion and warmth for myself, I will feel overcome with a sense of loss/grief	0	1	2	3	4
7.	I fear that if I become kinder and less self-critical to myself then my standards will drop	0	1	2	3	4
8.	I fear that if I am more self compassionate I will become a weak person	0	1	2	3	4
9.	I have never felt compassion for myself, so I would not know where to begin to develop these feelings	0	1	2	3	4
10.	I worry that if I start to develop compassion for myself I will become dependent on it	0	1	2	3	4
11.	I fear that if I become too compassionate to myself I will lose my self-criticism and my flaws will show	0	1	2	3	4
12.	I fear that if I develop compassion for myself, I will become someone I do not want to be	0	1	2	3	4
13.	I fear that if I become too compassionate to myself others will reject me	0	1	2	3	4
14.	I find it easier to be critical towards myself rather than compassionate	0	1	2	3	4
15.	I fear that if I am too compassionate towards myself, bad things will happen	0	1	2	3	4
